# The G7 Summit 2021: time for our world leaders to step up to the challenge of antimicrobial resistance

**DOI:** 10.1099/acmi.0.000298

**Published:** 2021-12-14

**Authors:** Lovleen Tina Joshi

**Affiliations:** ^1^​ School of Biomedical Sciences, University of Plymouth, Drake Circus, Plymouth PL4 8AA, UK

**Keywords:** antimicrobial resistance, climate change, COVID-19, G7, pandemic, policy

In June 2021, I wrote a strongly worded opinion piece published in the *New Statesman* about the G7 Summit and antimicrobial resistance (AMR) [1]. The piece highlighted that AMR should be addressed in the G7 Summit agenda and that our world leaders must recognize that research and investment into feasible solutions are urgently needed. This personal view adds to that original article.

The G7 Summit took place in Carbis Bay, Cornwall, UK on the 11–13 June 2021 and the UK took Presidency of the G7 group of nations [2]. The G7 comprises seven member countries, namely the UK, the USA, Canada, Japan, Germany, France and Italy, as well as the EU (a ‘non-enumerated member’). The summit represented over 60 % of people living in the democratic world, i.e. over half the world’s economy [3]. In numbers, that is 2.2 billion people being represented by 11 leaders making serious decisions around 1 table in Cornwall. The theme was Build Back Better. The main priority during the UK’s presidency is ‘leading the global recovery from coronavirus while strengthening our resilience against future pandemics’ [4].

Antimicrobial resistance (AMR, or ‘antibiotic resistance’ when referring to bacteria) is an insidious and silent pandemic that is already happening. It is not a future pandemic and the microbiology community are well aware of this. For those unfamiliar with AMR, it is one of the most urgent global healthcare challenges for the human species, alongside climate change. Micro-organisms such as fungi, parasites, bacteria and viruses that can cause infections have evolved and are evolving to become resistant to the antimicrobial drugs that we use to treat them. Overuse of antimicrobials in healthcare, animals and the environment has significantly accelerated AMR globally [5]. The latest figures suggest that AMR will cause over 10 million deaths per year by 2050, which is more than deaths from cancer and diabetes combined, and more than the current coronavirus disease 2019 (COVID-19) death toll of 4.5 million deaths worldwide since 2019 [6, 7]. The issue of AMR was covered in depth at the G7 Summit, specifically in the G7 Carbis Bay Health Declaration and in detail within the G7 Communique by the health ministers released on 4 June 2021 [8, 9]. The Communique in particular makes frequent reference to tackling AMR specifically in a One Health context and is generally a progressive and positive policy document acknowledging AMR and highlighting potential solutions [9]. This is a must read for those interested in AMR developments, solutions and policy. A One Health approach to AMR is paramount for implementing effective solutions, which include (but are not limited to) antimicrobial discovery, vaccines, diagnostics, stewardship and infection control and prevention (IPC) [6].

The main approach to tackle AMR has relied on antimicrobial discovery and development. While there is merit to this approach, the antibiotic discovery economic pipeline is *still* broken and this requires investment and long-term planning to address effectively. A recent World Health Organization (WHO) report indicated that ‘none of the 43 antibiotics in development target the most antibiotic resistant bacteria’, which is worrying long term [10]. However, the G7 health ministers discussed the importance of subscription models for antibiotic use as a possible solution [9]. The UK is currently trialling this subscription model for antimicrobial use, with highly anticipated results [11]. Microbial evolution of resistance to antimicrobials does not appear to be acknowledged in documents, however. This is important to note, as we must stay ahead of micro-organisms that are developing antimicrobial resistance by producing more new antimicrobial drugs. Alternative approaches include looking at vaccines as a targeted solution to prevent AMR infections [10]. However, if we are truly learning lessons from the COVID-19 pandemic, then there is the obvious topic of overcoming vaccine hesitancy and increasing vaccine confidence, which the G7 have also acknowledged [9, 12].

Diagnostics, however, are a feasible alternative solution to tackle AMR by enabling clinicians to rationalize critical antimicrobial use and increase antimicrobial stewardship. However, there has been limited global funding to accelerate diagnostics development for AMR infections [13]. The development of innovative technologies to detect antibiotic-resistant infections is critical to help preserve our last lines of working antibiotics. Improving antibiotic prescribing practice by encouraging the development of new innovative diagnostics is key within the UK Five Year AMR National Action Plan and the AMR review written by Lord O’ Neill in 2014 [6, 14]. Currently, patients who show signs of bacterial infection are given empirical antibiotic medication (broad-spectrum antibiotics) to ensure that the disease does not progress [15]. Clinical samples from the infective site are transported to hospital laboratories, either from GP surgeries or from hospital wards, which can affect sample stability and the reliability of results. Laboratory testing consists of microbiological culture, antimicrobial susceptibility disc testing and rapid PCR testing in algorithm, all of which require resources and time (2–3 days) to generate accurate results [6, 16, 17]. In the UK, 70–80 % of all antibiotics are prescribed in the community and 23 % of these are thought to be unnecessary [18, 19].

Current diagnostic tests employed at point of care often rely on the detection of inflammatory markers, such as C-reactive protein, for indication of bacterial and viral infections [20, 21]. While these are now becoming widely adopted across the NHS, there are issues with the appropriateness of using non-specific inflammatory markers (which do not differentiate between co-morbidities, such as arthritis) to specifically detect bacterial infection and aid stewardship [22].

Hence, investment into novel diagnostics for AMR, ideally for use at point of care, could help to rapidly diagnose patients with AMR infections without relying on empirical methods, assist in tailoring effective antibiotic treatment regimens and promote antibiotic stewardship. Rapid identification of AMR infection would reduce patient mortality, transmission of infection and costs to healthcare providers. The benefits of diagnostics as an alternative approach are huge. There is also an urgent need to develop low-cost and portable diagnostics for use in low-to-middle income countries (LMICs), where access to medical care is limited and patient suffering/mortality is high [17].

Another approach is infection prevention and control (IPC). IPC solutions in the context of AMR appear to have been overlooked in recent years despite their obvious and important links to water sanitation and hygiene (WASH) [23]. However, in the G7 health ministers’ Communique, IPC is noted as being essential to addressing AMR effectively: ‘The pandemic also highlighted the importance of infection prevention and control (IPC) measures to tackle AMR, targeting both healthcare-associated and community-associated infections’ [9]. IPC is at the core of preventing pathogens from transmitting to patients, thus preventing acquisition and subsequent infection in healthcare settings and within the community. Education surrounding hand hygiene has increased in relation to the COVID-19 pandemic, but there appears to be a public misconception that hand sanitizer is the main way of preventing transmission of infections. There is still a gap in public understanding that hand washing with soap and water is the best way of removing micro-organisms to prevent infection [24]. There are several reasons that IPC has previously been overlooked: partly due to assumptions that current IPC measures are fit for purpose for decontamination of all AMR micro-organisms, that biocides/disinfectants are working effectively in healthcare settings, that emerging biocide resistance is not an issue and that hygiene and sanitation are larger infrastructure issues to be dealt with by policymakers/governments [24, 25].

In LMICs the burden of AMR is high, partly because of the lack of access to healthcare facilities, infrastructure and WASH. If we consider that the world population is predicted to increase to 9.7 billion by 2030, that also puts additional strain on sanitation infrastructure in highly populated countries [26]. The majority of faeces/sewage produced by human populations is released into water without treatment [5]. This has led to increased persistence of AMR genes across the environment and and has contributed to AMR gene movement across ocean currents. This gene dissemination has been further impacted on by climate change and global temperature rise; AMR genes have even been found in the Arctic [27, 28]. The key thing for our world leaders to realize is that these global health challenges impact on one another significantly.

An important point to note here is that UK Overseas Development Aid cuts have severely affected the AMR research community globally, and many collaborative UK research projects in LMICs have had to be abandoned [29]. Finally, education still presents as the most obvious way of preventing overuse of antimicrobials. By informing the public, healthcare professionals and world leaders, we can highlight that antimicrobials are a finite, precious resource that needs to be protected and used sparingly.

The outcome of the 2021 G7 Summit has actually been hopeful for AMR. While we still have significant hurdles to overcome in developing sustainable solutions to tackle AMR long term, the acknowledgement of AMR within the G7 documents is an important step forward. Investing in multiple solutions is perhaps the best strategy to ensure we stay ahead of the microbes. Solutions such as antimicrobial development pipelines, subscription models and alternatives such as AMR stewardship, vaccines, clinical education, diagnostics and IPC all require research and development. It is said that ‘the proof of the pudding is in the eating’; well I, amongst others in the AMR sphere, are pretty interested in what that pudding tastes like! Will the resilience strategy survive the true test of AMR? Only time will tell.
